# A population-based study of neonatal air transport in the Arctic region of Norway from 1994 to 2023

**DOI:** 10.3389/fped.2025.1594729

**Published:** 2025-07-01

**Authors:** Lene Nymo Trulsen, Lisa Gullhav Hansen, Nils Thomas Songstad, Astri Lang, Claus Klingenberg

**Affiliations:** ^1^ Department of Paediatrics and Adolescent Medicine, University Hospital of North Norway, Tromsø, Norway; ^2^ Research Group for Child and Adolescent Health, Faculty of Health Sciences, UiT-The Arctic University of Norway, Tromsø, Norway; ^3^ Neonatal Department, Oslo University Hospital, Oslo, Norway

**Keywords:** transport, prematurity, congenital malformations, hypothermia, respiratory management

## Abstract

**Background and aims:**

Regionalized centralization of moderate and high-risk pregnancies is essential, but a well-organized postnatal transport service is equally important. This study evaluates the overall activity and clinical outcomes of the neonatal air transport team (NATT) at the University Hospital of North Norway (UNN) in Tromsø, covering a large catchment area in the Arctic region of Norway.

**Methods:**

Medical data from all neonatal air transports between the years 1994–2023 were recorded prospectively. Body temperature, blood glucose and blood gas within 3–6 h after arrival at UNN were assessed from medical files retrospectively. To assess temporal changes, we compared data between 1994 and 2008 (Period 1) and 2009–2023 (Period 2).

**Results:**

A total of 882 acute transports were included. Of these, 655 (74.3%) were referrals to the tertiary neonatal unit at UNN and 227 (25.7%) transfers to national surgical centers. Most transports (61.5%) were by fixed wing aircrafts. The proportion of infants transported due to congenital heart defects (CHD), prematurity and asphyxia was lower in Period 2. When comparing Period 1 and 2, upon arrival we found similar rates of hypothermia (9.8% vs. 6.7%, *p* = 0.17) and hypercapnia (17.3% vs. 15.3%, *p* = 0.55), but decreasing rates of hypocapnia (6.7% vs. 2.5%, *p* = 0.014) and hypoglycemia (10.8% vs. 2.3%, *p* = 0.001). There were low rates of outborn very low birth weight (VLBW) infants (<1,500 g) in both periods; 4.3% and 3.1%. However, severe IVH was observed in 6/29 (20.7%) outborn VLBW-infants vs. only 21/356 (5.9%) inborn VLBW-infants in the last 15-year period.

**Conclusion:**

Decreasing rates of transport due to prematurity and CHDs is probably secondary to improved perinatal care. Rates of hypoglycemia and hypocapnia improved in the second 15-year period, but further focus on improvements in both temperature and CO_2_ control is warranted. Acute transport of VLBW-infants is associated with a markedly increased risk of severe IVH. In-utero transfer of women with threatened preterm birth to a tertiary perinatal center is therefore paramount.

## 1 Introduction

Large changes have occurred within antenatal and perinatal care over the last three decades. Improved antenatal ultrasound has led to a higher level of detection of congenital malformations (1). The centralization of preterm deliveries and other high-risk pregnancies has improved perinatal outcomes in many countries (2, 3). In particular, very preterm infants and infants with congenital malformations benefit from being born at tertiary centers with access to specialized medical teams and immediate advanced intensive care (3, 4). Pregnancies with increased risk of perinatal (e.g., obesity) and/or neonatal complications (e.g., maternal diabetes) have also increasingly been selected to deliver in hospitals offering specialized obstetric and neonatal care (5).

Although centralization of specialized medical care may improve outcomes at high volume centers, it comes at the expense of medical experience and skills at smaller health institutions. Infants born at local hospitals where specialized neonatal care is not available and who suffer unexpected complications during delivery, have unrecognized congenital conditions or are born very preterm will be in need of stabilization and safe transport to regional centers (6). A well-organized postnatal transport service is therefore an increasingly important component of the health care service (7, 8).

Transporting sick newborns presents unique challenges. Temperature regulation may be challenging, particularly in low birthweight infants and in the setting of low ambient air temperatures (9). Environmental factors such as noise, air pressure and vibration within the transport environment may disrupt physiological stability in fragile infants (10–12). Finally, the ability to monitor and intervene on clinical complications occurring during transport are more limited during transport than within the neonatal intensive care unit (NICU) environment. Access to specialized transport equipment and a dedicated transport team with sufficient experience and training is therefore essential in ensuring safe neonatal transport (13). Today, it is possible to deliver advanced respiratory support during transport, and other improvements in monitoring, temperature control and security have emerged.

The sparsely populated Arctic region of Norway present unique challenges with large distances between health care institutions, a rough Arctic climate and only one tertiary care neonatal unit for the whole region located in Tromsø. In this region surfactant therapy for respiratory distress syndrome became available in the early 1990s. In 2007 therapeutic hypothermia was introduced for infants with moderate-severe hypoxic ischemic encephalopathy (HIE). A fetal medicine service in Tromsø was built up from the late 1990s and routine newborn pulse oximetry screening was introduced in this region in 2009.

The primary aim of this study was to evaluate the overall quality and outcomes of neonatal transports in the northernmost region of Norway between 1994 and 2023. We secondly aimed to evaluate changes that have occurred along with improved perinatal care, by comparing outcomes in the first and the last 15-year period of the 30-year study period, particularly focusing on very low birth weight (VLBW) infants and infants with congenital heart defects.

## 2 Material and methods

### 2.1 Study design

This is a retrospective audit of all acute neonatal air transports conducted by the neonatal air transport team (NATT) at the University Hospital of North Norway (UNN) in Tromsø between January 1994 and December 2023—a 30-year period.

### 2.2 Setting

**Figure 1:**
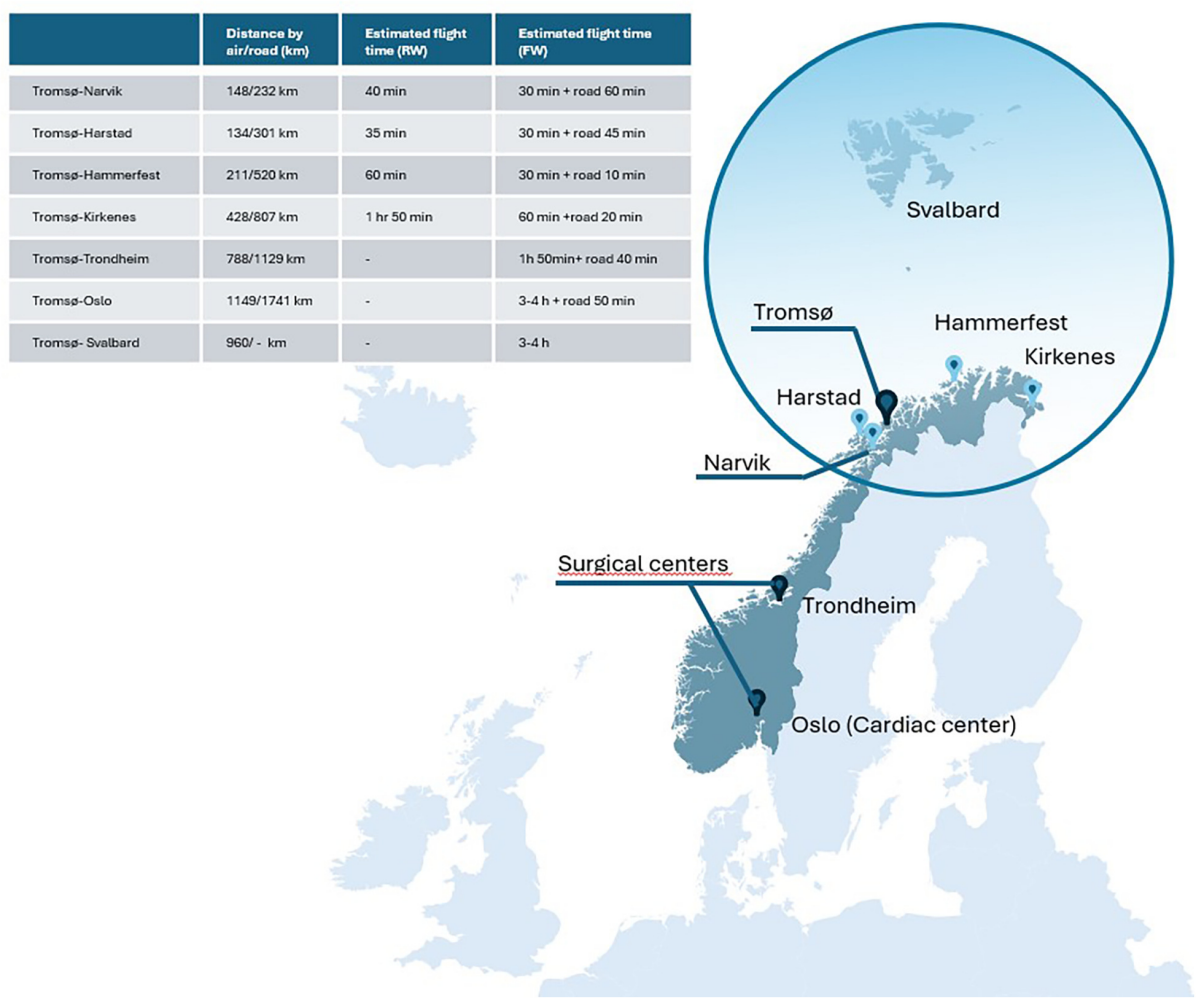
Catchment area of the transport team and distances to tertiary surgical centers (map of Norway) light blue dots are local hospitals (level 1–2).

Troms and Finnmark are the two northernmost counties of Norway, located within the Arctic region of Norway (14). The region has an area of around 75,000 km^2^, equivalent to 23% of Norway's total area (Figure 1), and larger than the Republic of Ireland. It is a sparsely populated region with a population of only 245,000 inhabitants (2024 census). The annual birth rate in the catchment area has declined from 3,583 in 1994 to 2,236 in 2023 (15). Despite declining birth rates, the number of delivery units (*n* = 8) has remained unchanged over the last 30 years. Of these eight, 3 are midwife-managed maternity units (level 1), four are maternity units at local hospitals (level 2) and the last is a tertiary obstetric department at the University Hospital of North Norway (UNN) in Tromsø. The region hosts two pediatric departments, of which UNN is the only hospital with a tertiary level NICU treating very preterm infants down to 23 weeks gestation and other infants in need of mechanical ventilation/intensive care. In Norway neonatal surgery is centralized to Trondheim or Oslo, and neonatal cardiac surgery to Oslo.

In 2012 national guidelines were implemented defining at which level (1 to 3) pregnant women were recommended to deliver according to existing risk factors and selection criteria (5). These guidelines stated that all high-risk deliveries including extremely preterm deliveries, severe pre-eclampsia and severe obesity should take place at level 3 institutions. Furthermore, prenatal transfer of fetuses diagnosed with complex congenital heart defects and/or other severe congenital malformations (e.g., abdominal wall defects and diaphragmatic hernia) to neonatal surgical centers in Trondheim or Oslo has increasingly been performed over the last 10–20 years.

### 2.3 Transport team and equipment

**Figure 2:**
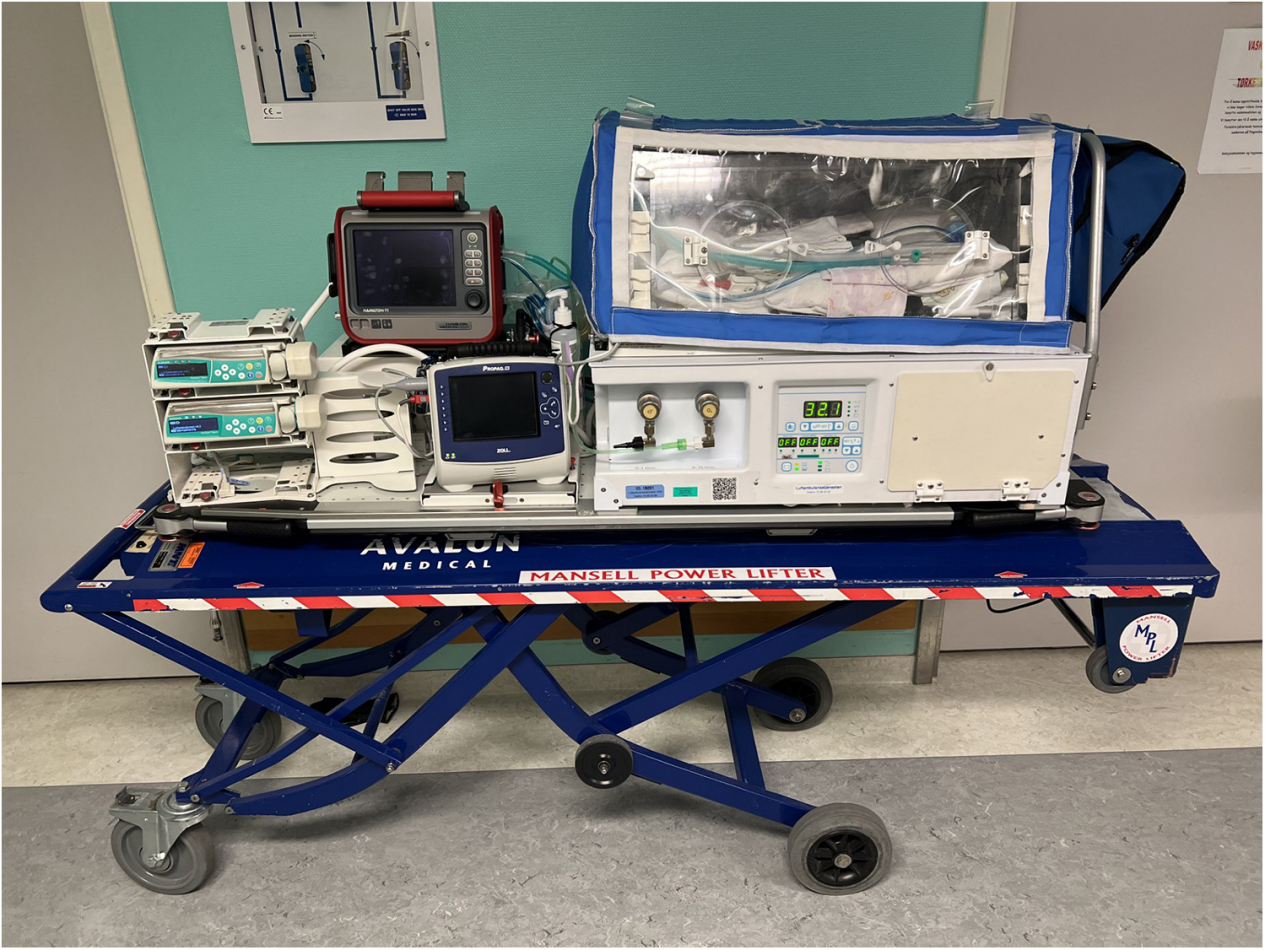
Airborne transport incubator with Hamilton T1 ventilator and current setup with patient monitor, oxygen cylinder and infusion pumps outside the incubator. Note a covering blanket to prevent heat loss during cold outside temperature.

Large distances within the Troms and Finnmark region imply that virtually all neonatal transports are conducted by air, either by rotary wing (RW) or fixed wing (FW) ambulance aircrafts. The only road transports were the transfers from hospitals to/from airports. During the entire study period the NATT at UNN has consisted of a NICU-nurse and a certified pediatrician, but since 2011 the pediatricians involved have been a trained neonatologist who work in the NICU on a regular basis. The quality of the transport equipment has evolved during the 30-year period. From 1994 to 2008 the NATT used a Drager incubator with a Babylog Plus ventilator with no trigger function and limited options to provide continuous positive airway pressure (CPAP). From 2008 to 2017 we used a Mansell Neocot transport incubator and a Bio-Med Crossvent ventilator. Since 2017 we have used the Airborne Life transport incubator and a Hamilton T1 neonatal transport ventilator, providing the option for synchronized ventilation with an adequate humidifier (Neo-Pod T, International Biomedical, Texas, USA), non-invasive respiratory support (CPAP and nasal high flow therapy), but not high frequency ventilation (Figure 2). Since 2008 we have offered active cooling during transport of selected infants fulfilling clinical criteria for therapeutic hypothermia. Active cooling has been provided with plastic gloves filled with cold water and placed in both flanks of the infants, if infants were considered stable. Servo controlled cooling has not been and is still not available for transport. Continuous rectal temperature probes are employed during most transports, and since 2008–2009 we have aimed to use end tidal CO2 (EtCO_2_) measurement in all ventilated infants. If tolerated, enteral feeds by the nasogastric route are routinely provided during air transport. Infants not tolerating enteral feeds receive glucose maintenance infusion during transport. Parental presence during transport has varied, depending on space and size of the aircraft, but was not routinely registered for this study.

### 2.4 Data collection and variables

Baseline data concerning the medical problem at referral, mode of transport, stabilizing procedures prior to transport and any clinical or technical problems occurring during transport are recorded prospectively in a “transport book” for all infants transported by the NATT *to* or *from* UNN. We retrospectively extracted data from patient medical files regarding place of birth, birth weight (BW), Apgar score, gestational age (GA), body temperature, blood glucose and blood gases on arrival, final diagnoses and outcomes. Hypothermia was defined as temperature *<*36.0℃, hyperthermia as temperature *>* 37.5℃ (8), hypoglycaemia as blood glucose *<*2.2 mmol/L (40 mg/dl), hypocapnia as pCO2 *<* 4.0 kPa (30 mm Hg), hypercapnia as pCO2 *>* 7.3 kPa (55 mm Hg) and acidosis as pH *<* 7.25 (16). Background demographic data were obtained from the Medical Birth Registry of Norway (MBRN) (15). Data on all VLBW-infants (<1,500 g) within the region were collected from the neonatal database at UNN, as all these infants are cared for in this tertiary NICU. Intraventricular hemorrhage (IVH) grade III-IV was defined according to the criteria of Papile (17). The dataset was merged with data from a previously published paper covering the study period 1994–2003 (18). The study was approved by the data protection officer at the University Hospital of North Norway.

### 2.5 Statistical analysis

Variables are reported as median and interquartile range (IQR). To compare differences between the two 15-year periods (Period 1; 1994–2008 and Period 2; 2009–2023), we used non-parametric tests for continuous variables and chi-square or Fisher's exact test for categorical variables, as appropriate. All analyses were two-tailed and *p* *<* 0.05 was significant. Statistical analysis was performed with SPSS for Windows software version (2025).

## 3 Results

### 3.1 Overview of all transports

Between January 1994 and December 2023, a total of 1197 neonatal transports were performed by the NATT. Of these 882 (74%) were classified as acute transports, of which 655 (74.3%) were acute referrals of newborns born within the region to the tertiary NICU at UNN and 227 (25.7%) transfers from UNN to national surgical centers (Table 1). The number of acute transports performed by the NATT increased from 398 in Period 1 to 484 in Period 2. The remaining 315 (26%) transports were elective step-down transfers from UNN to local hospitals, and data from these are not included in further analysis.

**Table 1:** Transport overview and patient characteristics.

	Total *n* = 882	1994–2008 *n* = 398	2009–2023 *n* = 484	*P* value^a^
Transport type				
Acute referrals to UNN	655 (74.3)	293 (73.6)	362 (74.8)	0.69
Transfers to national surgical centers	227 (25.7)	105 (26.4)	122 (25.5)	0.69
Transport platform				
Fixed wing aircraft	542 (61.5)	260 (65.3)	282 (58.3)	**0**.**03**
Rotary wing aircraft	326 (37.0)	135 (33.9)	191 (39.5)	0.09
Both fixed and rotary wing aircraft	12 (1.4)	3 (0.8)	9 (1.9)	0.16
Patient data				
Gestational age (weeks)	39 (35–40)	38 (35–40)	39 (35–40)	**0**.**022**
Birth weight (grams)	3,225 (2,480–3,721)	3,152 (2,333–3,635)	3,338 (2,590–3,800)	0.08
Birth weight < 1,500 grams^c^	80 (9.1)^c^	34 (9.6)	46 (8.7)	0.62
5-minute Apgar scores	9 (7–10)	9 (7–9)	9 (7–10)	0.15
Transport diagnosis^b^				
Unspecified respiratory distress and “other”	287 (32.5)	88 (22.1)	199 (41.1)	**<0**.**001**
Congenital heart disease (CHD)	148 (16.7)	87 (21.9)	61 (12.6)	**<0**.**001**
Prematurity	135 (15.3)	74 (18.6)	61 (12.6)	**0**.**024**
Infection	110 (12.5)	43 (10.8)	67 (13.8)	0.13
Other malformations than CHD	101 (11.5)	48 (12.1)	53 (11.0)	0.73
Asphyxia	101 (11.5)	58 (14.6)	43 (8.9)	**0**.**013**

^a^ *P*-values by chi-square test.

^b^ Transport diagnosis were not always equal to final diagnosis at discharge, in particular for the group Unspecified respiratory distress and “other”, which included predominantly different kinds of respiratory conditions (wet lung, transient tachypnea), but also various diagnoses like hyperbilirubinemia, hypoglycemia or seizures.

^c^ Includes infants with birth weight < 1,500 g referred to UNN (*n* = 43) or transferred for evaluation/management at surgical centers (*n* = 37).

Data are presented as frequencies, numbers (%) or median (IQR).

UNN, university hospital of North Norway; IQR, interquartile range.

Bold values indicate statistically significant results (*P* < 0.05).

Table 1 displays an overview of type of type of transport, transport platform and baseline patient data including transport diagnosis. Most (61.5%) acute transports were by FW-aircrafts, but the proportion of transports by FW-aircraft decreased from Period 1 to Period 2 (*p* = 0.003). The main indications for neonatal transports were “Unspecified respiratory distress/other diagnoses” (32.5%), suspected or proven congenital heart defects (CHD) (16.7%), prematurity (15.3%), infections (12.5%), perinatal asphyxia (11.5%) and other congenital malformations (11.5%). Some overlap occurred within these categories since preterm infants often received respiratory support. We observed a reduction in the proportion of transports due to CHD, asphyxia and prematurity between Period 1 and 2 (Table 1).

All stable patients requiring surgery are usually transferred to surgical centers, if possible. During the last 15-year period, 11 very preterm infants have been operated for intestinal injuries (necrotizing enterocolitis/focal intestinal perforation) as they were considered too unstable for transport. Surgery has then been performed by gastrointestinal surgeons at UNN.

### 3.2 Transport interventions and outcomes

Table 2 displays medical interventions during transport and the primary outcomes of interest. Between Period 1 and 2, the use of mechanical ventilation during transport decreased, and concomitantly the use of CPAP increased. Hypothermia upon arrival in the NICU was observed in 8.1% of cases and hyperthermia in 11.7% of cases, with no differences between the two periods. In Period 2 we observed a significant reduction in the number of infants with hypocapnia upon arrival in the NICU. Similarly, the rate of hypoglycemia upon arrival in the NICU also decreased from Period 1 (10.8%) to Period 2 (2.3%). Transport-related mortality, defined as death within 24 h upon arrival, was 0.9% for the 30-year period, with only two transport-related deaths occurring in Period 2. Causes of early deaths were severe perinatal asphyxia, CHD and extreme prematurity. The proportion of infants who died before discharge from the NICU decreased significantly in Period 2.

**Table 2:** Transport interventions, quality metrics and outcomes.

	Total *n* = 882	1994–2008 *n* = 398	2009–2023 *n* = 484	*P* value^a^
Medical interventions during transport				
Mechanical ventilation	193/882 (21.9)	108/398 (27.1)	85/484 (17.6)	**<0**.**001**
Continuous positive airway pressure	105/882 (11.9)	18/398 (4.5)	85/484 (17.6)	**<0**.**001**
High-flow nasal cannula	8/882 (0.9)	None	8/484 (1.6)	
Prostaglandin E1 infusion	69/882 (7.8)	36/398 (9.0)	33/484 (6.8)	0.22
Pleural drainage	16/882 (1.8)	6/398 (1.5)	10/484 (2.1)	0.54
Clinical data on arrival at UNN^b^				
Temperature (°C)	36.9 (36.5–37.3), *n* = 568	36.9 (36.5–37.3), *n* = 254	36.9 (36.6–37.3), *n* = 314	0.79
Hypothermia (<36.0°C)^c^	45/554 (8.1)	25/254 (9.8)	20/300 (6.7)^c^	0.17
Hyperthermia (>37.5°C)	65/554 (11.7)	26/254 (10.2)	39/300 (13.0)^c^	0.31
pH < 7.25	40/474 (8.4), *n* = 474	13/232 (5.6), *n* = 232	27/242 (11.2), *n* = 242	0.79
pCO_2_ (kPa)	5.6 (5.0–6.7), *n* = 467	6.1 (5.0–6.8), *n* = 225	5.6 (4.9–6.6), *n* = 242	0.26
Hypocapnia < 4.0 kPa (40 mm Hg)	21/467 (4.5)	15/225 (6.7)	6/242 (2.5)	**0**.**014**
Hypercapnia > 7.3 kPa (55 mm Hg)	76/467 (16.3)	39/225 (17.3)	37/242 (15.3)	0.55
Blood glucose (mmol/L)	4.0 (3.2–5.0), *n* = 489	3.8 (2.8–4.8), *n* = 223	4.1 (3.4–5.1), *n* = 266	**0**.**008**
Hypoglycemia < 2.2 mmol/L (40 mg/dl)	30/532 (5.6)	24/223 (10.8)	6/266 (2.3)	**0**.**001**
Short- and long-term mortality				
Death during or within 24 h of transport	8 (0.9)	6 (1.5)	2 (0.4)	0.088
Death before discharge from the NICU	73 (8.3)	53 (13.3)	20 (4.1)	**<0**.**001**

^a^ *P*-values by chi-square test or non-parametric test, as appropriate.

^b^ Clinical data on arrival UNN based on 655 acute referrals to UNN. Denominator varies due to missing data, e.g., blood gas not obtained after arrival in many cases that were considered in a stable condition upon arrival.

^c^ Fourteen patients with severe perinatal asphyxia and actively initiated therapeutic hypothermia during transport are excluded.

Data are presented as frequencies, numbers (%) or median (IQR).

UNN, university hospital of North Norway, IQR; interquartile range, NICU; neonatal intensive care unit.

Bold values indicate statistically significant results (*P* < 0.05).

### 3.3 Very low birth weight infants

During the study period there were 782 live-born VLBW-infants admitted from the catchment area of the tertiary NICU in Tromsø. Of these, twenty-nine VLBW-infants were outborn and were transported by the NATT to UNN within the first 48 h of life; 17/392 (4.3%) in Period 1 and 12/390 (3.1%) in Period 2, *p* = 0.35. Median (IQR) time for commencement of patient transport was 2 (2–3) hours of life. Severe IVH was observed in 6/29 (20.7%) outborn VLBW-infants transported in first 48 h of life. In contrast, the rates of severe IVH among inborn VLBW-infants in our NICU in the last 15-year period was 21/356 (5.9%). Among the acutely retrieved VLBW-infants, 2/17 (11.8%) died within the first 24 h after transport in Period 1 and 2/12 (16.7%) in Period 2. Hypothermia rates on arrival among VLBW infants who were acutely transported within the first 48 h of life were high, both in Period 1 (6/17; 35.3%) and in Period 2 (5/12; 41.7%). In contrast, the rates of hypothermia among inborn VLBW-infants in our NICU in the last 15-year period was 54/356 (15%). There were 51 other transports with VLBW-infants, mainly for evaluation or treatment to neonatal surgical/cardiac centers or referrals to UNN for other reasons. Postnatal age for these transports were median (IQR) 54 (30–87) days.

### 3.4 Congenital heart defects

Sixty-four infants with CHD were transferred for neonatal cardiac evaluation/surgery. The most common cardiac diagnoses were coarctation of the aorta (CoA) (15/64; 23.3%), transposition of the great arteries (TGA) (11/64; 17.2%), and hypoplastic left heart syndrome (HLHS) (7/64; 10.9%). The proportion of acute transports related to CHD declined significantly from 21.9% in Period 1 to 12.6% in Period 2 (Table 1). Median (IQR) age at transport was 3 (1–11) days in Period 1 and 2 (0–8) days in Period 2, *p* = 0.38. There were no observed differences in the proportion of infants receiving prostaglandin E1 (PGE_1_) infusion between the two periods. More than 70% of PGE_1_ infusions were provided during transport of infants in their first 3 days of life in both periods. In Period 1 vs. Period 2 significantly more infants with PGE_1_ infusion were mechanically ventilated during transport (28/36 vs. 13/33, *p* = 0.002).

## 4 Discussion

### 4.1 Main findings

This study provides a population-based analysis of nearly 900 acute neonatal air transports conducted by the NATT at the University Hospital of North Norway over a 30-year period. The NATT operates within a geographically large but sparsely populated region of Norway, and under arctic climatic conditions. The audit demonstrates a relative decrease in neonatal transports due to prematurity, perinatal asphyxia and CHD in the last 15-year period. A reduction in the use of mechanical ventilation, rates of hypoglycemia, hypocapnia and a low transport related mortality was also observed in in the last 15-year period. However, some challenges when performing neonatal transport within the arctic settings persists, particularly with regard to temperature regulation.

Despite reduced birth rates within the region, the number of acute neonatal transports was higher in the second time-period than in the first. Proportionately fewer transports were due to prematurity, CHD or perinatal asphyxia. On the other hand, the proportion of babies classified with “Unspecified respiratory distress/other diagnoses” was significantly higher. Many factors likely contribute to these changes. The quality of prenatal care and particularly fetal medicine is markedly improved over the 30 years covered in this study. An increased proportion of fetuses with CHD or other surgical malformations are prenatally identified and pregnancies centralized to the right level of care before birth (19, 20). The introduction of stricter selection criteria for high-risk pregnancies in 2012 is also likely to have reduced the risk for adverse obstetric events/perinatal asphyxia at level 1 and 2 delivery units. On the other hand, increased centralization and improved standards for neonatal care have in combination influenced the ability and experience of smaller delivery units to take care of infants with less severe neonatal morbidities after birth. This may have increased the demand for postnatal transport of less severe conditions.

### 4.2 Changes in short term clinical outcomes

Compared to the first 15-year time-period, rates of hypoglycemia and hypocapnia were lower in Period 2. A reduction in use of mechanical ventilation during transport along with improved access to EtCO_2_ measurement have probably reduced the risk for inadvertent hyperventilation. After the publication of data from the first 10 years of neonatal transport where rates of hypoglycemia were relatively high (18), an increased awareness within the NATT staff may be a reason for the low rate of hypoglycemia upon arrival in the last 15-year period.

Advancements in transport incubators and ventilation support have over time facilitated substantial improvements in neonatal care during transport. The transition from older ventilators to the Hamilton T1 ventilator in 2017 with the possibility of providing volume-targeted ventilation and humidified gas has enhanced the quality of respiratory support. Additionally, increased training and experience among the members of the transport teams may have contributed to better stabilization and reduced need for invasive respiratory support. This is in line with findings from a transport services in Western Australia were neonatal specialist teams experienced less unintended events during transports compared to non-neonatal specialist teams (21). A shift to more non-invasive respiratory support, particular among preterm infants, may also explain the reduced need for mechanical ventilation in period 2.

Hypothermia is a known risk factor for neonates during transport (16, 22, 23) and a quality metric of neonatal transport care (24–26). Despite overall improvements in skill and equipment, neonatal transports under arctic conditions continue to pose challenges regarding temperature control. Around 8% of all infants arrived with hypothermia, and among VLBW-infants around 4 in 10 were hypothermic upon arrival. A study from India found a rate of hypothermia on arrival of 8.6% in their cohort study under warmer conditions (27), whereas a study from the Madrid Newborn Transport team who used a higher cut-off (<36.5℃) found a prevalence of hypothermia of 33.4% (26). Low gestational age and mechanical ventilation are known risk factors for hypothermia after transport (22). Another possible risk factor of hypothermia is infusion of cold solutions from outside the incubator, particularly in the smallest babies. This is one aspect of the incubator setup that may be amenable to improvement in terms of temperature regulation. Our data may suggest that the transport incubators setup currently used are insufficient in regulating body temperature in the smallest infants during long transports and in low ambient temperatures, indicating a need for improvement.

### 4.3 Very low birth weight infants

The vast majority (96%) of VLBW-infants born within the Troms and Finnmark region during the last 30 years were delivered at UNN. Our low rate of outborn VLBW-infants delivery compares favorably with data from other countries which report rates ranging from 2%–37% (3, 28–32). A large cohort study from Japan reported a rate of 37% outborn preterm transferred to tertiary hospitals (32). We speculate that caregivers have a high awareness of the risks of preterm delivery outside a NICU in our sparsely populated region. Thus, there seems to be a strong focus on timely transport of women with threatening preterm birth to the appropriate level of care. This is in line with what was seen in Finland, with a stable low rate of postnatal transfers of very preterm infants (2%–4%) after implementation of centralization guidelines in 2010. The mortality rate for outborn VLBW infants was low in our study, but a relatively high proportion suffered IVH grade III–IV, in line with previous studies (33, 34).

### 4.4 Congenital heart defects

Evaluating the epidemiological changes in transport of CHD babies during this 30-year period is challenging. On one hand, birth rates in the catchment region has declined. Thus the 30% reduction in babies with CHD transported reflects the reduction in birth rates. Concomitantly fetal medicine service has gradually increased prenatal diagnosis (35) and prenatal transfer to the national cardiac center in Oslo. Early postnatal diagnosis of duct dependent CHDs has also improved since pulse oximetry screening was established in our region in 2009 (36). In our study a lower proportion of transports were due to CHD in the last 15-year period. However, those transported to the national center for cardiac surgery had very severe heart malformations incl. TGA, CoA and HLHS and rates of PGE_1_-infusions were similar in the two periods. We did not record the PGE_1_ infusion doses, but early diagnosis may allow low-dose PGE_1_ to maintain ductal patency and is associated with a lower incidence of respiratory depression requiring mechanical ventilation (37). In line with this, we also found a significant reduction of CHD-babies receiving PGE_1_ infusion who were mechanically ventilated during transport in the last 15-year period. This aligns with our clinical impression that it is safe to transport babies spontaneously breathing when receiving a low-dose PGE1 infusion.

### 4.5 Areas for future improvements

The findings from this audit reveal several factors to further improve quality of neonatal transport in our region. Firstly, there is still a need for enhanced thermal management. Implementation of standardized protocols to prevent hypothermia and an increased focus among the NATT members may improve the standard of care, alongside with more efficient pre-transport warming strategies and improved incubator techniques. Secondly, a strong focus on respiratory support management to improve CO_2_-control during transport is important. During the study period, RW/FW-aircrafts of varying sizes and load capacities were used. The smaller aircrafts do not have the capacity to accommodate parents accompanying their newborn to the receiving unit. Strategies to facilitate parental presence during transport could improve both neonatal outcomes and the well-being of parents (38).

### 4.6 Strength and limitations

The strengths of this study include its long time span and a population-based coverage of all neonatal air transports in the region, limiting the risk of selection bias. Some data were prospectively recorded, but the study also has the inherent limitations associated with its partially retrospective design. Since data collection relies on medical record review, there were some missing or inconsistent data, potentially affecting accuracy. Data collection was also made by several investigators (LNT, AL, LGH) which may lead to variability in reporting. Changes and advances in neonatal care over time may have influenced outcomes, making it difficult to compare data across different time periods and to draw definitive conclusion about causation. Despite the limitations of this design, retrospective audits are of important value for quality improvements not only for the local team, but also for all other transport teams that face similar challenges and struggles of long distances and rough climate conditions.

## 5 Conclusions

The findings of this study demonstrate significant advancements in neonatal transport service in Northern Norway over the past three decades. Improvements in perinatal care, transport equipment and team expertise have contributed to a decline in transport related morbidity/complications and a very low transport related mortality. However, challenges still remain especially in optimizing thermal management and CO_2_-regulation during transport. Continued refinement of transport protocols and integration of new improved technologies will be crucial for the safety and efficacy of neonatal air transport in arctic conditions.

## Data Availability

The raw data supporting the conclusions of this article will be made available by the authors, without undue reservation.
